# Robotic hepaticojejunostomy training in novices using robotic simulation and dry-lab suturing (ROSIM): randomized controlled crossover trial

**DOI:** 10.1007/s00464-024-10914-8

**Published:** 2024-07-03

**Authors:** Julia E. Menso, A. Masie Rahimi, Maurice J. W. Zwart, Freek Daams, Joey de Hondt, Emir Karadza, Roberto M. Montorsi, Felix Nickel, H. Jaap Bonjer, Els J. M. Nieveen van Dijkum, Marc G. Besselink

**Affiliations:** 1 Amsterdam UMC, Department of Surgery, Location University of Amsterdam, Amsterdam, the Netherlands; 2 Cancer Center Amsterdam, Amsterdam, the Netherlands; 3 Department of Surgery, Amsterdam UMC, Location Vrije Universiteit, Amsterdam, the Netherlands; 4 Department of General, Visceral and Transplantation Surgery, Heidelberg University Hospital, Heidelberg, Germany; 5 Department of General, Visceral and Thoracic Surgery, University Medical Center Hamburg-Eppendorf, Hamburg, Germany; 6 Department of Surgery, Verona University Hospital, University of Verona, Verona, Italy

**Keywords:** Robotic surgery, Robotic suturing, Robotic simulation training, SimNow simulator, Training

## Abstract

**Background:**

Robotic suturing training is in increasing demand and can be done using suture-pads or robotic simulation training. Robotic simulation is less cumbersome, whereas a robotic suture-pad approach could be more effective but is more costly. A training curriculum with crossover between both approaches may be a practical solution. However, studies assessing the impact of starting with robotic simulation or suture-pads in robotic suturing training are lacking.

**Methods:**

This was a randomized controlled crossover trial conducted with 20 robotic novices from 3 countries who underwent robotic suturing training using an Intuitive Surgical^®^ X and Xi system with the SimNow (robotic simulation) and suture-pads (dry-lab). Participants were randomized to start with robotic simulation (intervention group, *n* = 10) or suture-pads (control group, *n* = 10). After the first and second training, all participants completed a robotic hepaticojejunostomy (HJ) in biotissue. Primary endpoint was the objective structured assessment of technical skill (OSATS) score during HJ, scored by two blinded raters. Secondary endpoints were force measurements and a qualitative analysis. After training, participants were surveyed regarding their preferences.

**Results:**

Overall, 20 robotic novices completed both training sessions and performed 40 robotic HJs. After both trainings, OSATS was scored higher in the robotic simulation-first group (3.3 ± 0.9 vs 2.5 ± 0.8; *p* = 0.049), whereas the median maximum force (*N*) (5.0 [3.2–8.0] vs 3.8 [2.3–12.8]; *p* = 0.739) did not differ significantly between the groups. In the survey, 17/20 (85%) participants recommended to include robotic simulation training, 14/20 (70%) participants preferred to start with robotic simulation, and 20/20 (100%) to include suture-pad training.

**Conclusion:**

Surgical performance during robotic HJ in robotic novices was significantly better after robotic simulation-first training followed by suture-pad training. A robotic suturing curriculum including both robotic simulation and dry-lab suturing should ideally start with robotic simulation.

**Supplementary Information:**

The online version contains supplementary material available at 10.1007/s00464-024-10914-8.

With the ongoing implementation of robot-assisted surgery, adequate training is in increasing demand and will mitigate the surgeon’s early learning curve [1–3]. Several studies aimed to create a standardized curriculum for robotic surgical education, but consensus on the optimal simulation training approach is still lacking [4, 5]. Various robotic training modalities have been investigated. On one hand, there are the Mimic dV-trainer and the da Vinci Skills Simulator (dVSS), both robotic simulation systems. On the other hand, there is the use of conventional dry and wet lab simulation with the da Vinci surgical system in inanimate or animal models, respectively [6, 7].

Robotic simulation improves robotic suturing performance [8], is transferable to the operating room, and can predict “live” performance [9]. Simulation training could contribute to maintenance and retention of robotic skills and adequately prepare future surgeons for robotic surgery [7]. Comparable outcomes between robotic simulation training and dry-lab simulation suggest that robotic simulation is a suitable alternative for dry-lab robotic training [6, 7]. Although dry-lab is suggested to be more feasible for practice of complex robotic tasks, it is also more expensive due to the use of robotic instruments and the robotic system [11, 12]. Previous comparative studies only assessed performance with basic robotic tasks and did not perform objective assessment of surgical performance with force measurements [8, 11, 13]. Robotic training with robotic simulation and dry-lab is most practical when the group is divided over both training modalities and thereafter cross over, as long as training value is similar between both sequences. However, evidence about the impact of training sequence is currently lacking. This is relevant when setting up curricula for robotic suturing training.

Hence, this study aims to determine whether the sequence of robotic suturing training, either starting with the SimNow simulator (robotic simulation) or with suture-pads (dry-lab), has a notable impact on the quality of robotic suturing as assessed both subjectively and objectively in artificial robotic hepaticojejunostomy (HJ). It is hypothesized that there is no difference between the two sequences and that robotic training could be given in a crossover setting.

## Materials and methods

### Study design

**Fig. 1:**
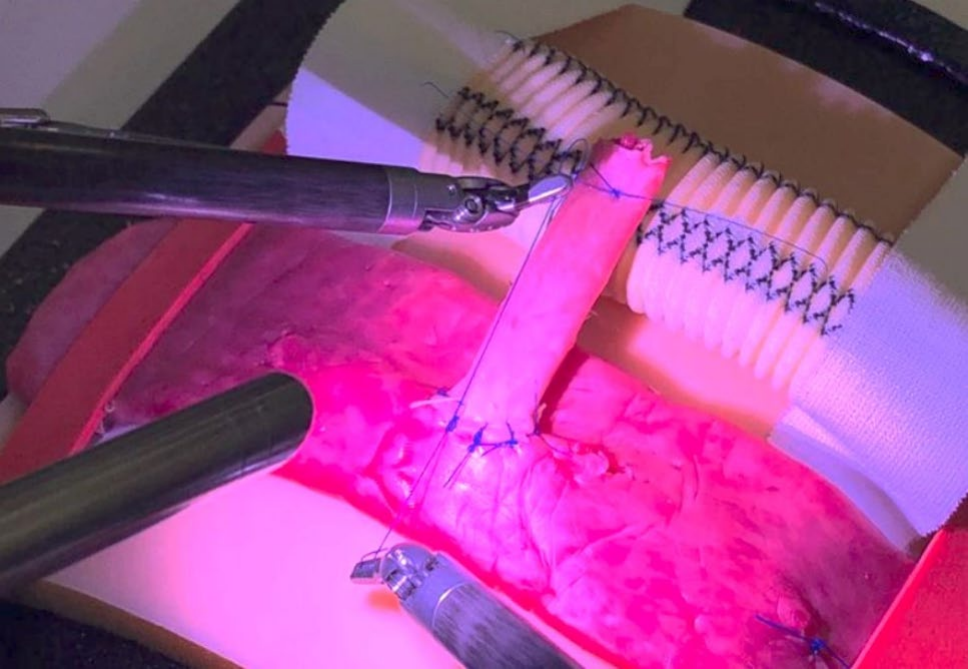
Hepaticojejunostomy with artificial organ models (biotissue)

We conducted a randomized controlled crossover trial with a non-inferiority design. All participants received robotic simulation training with the SimNow simulator and suture-pad training with the da Vinci X/Xi system (Intuitive Surgical Inc., Sunnyvale, California, USA). The participants were randomized to start either with robotic simulation (intervention group) or suture-pads (control group) and thereafter crossed over. After both training sessions, a robotic suturing exam followed: a HJ in biotissue (see Fig. 1). We used the CONSORT checklist when writing our report [14]. All procedures were performed at the Amsterdam Skills Centre and the Amsterdam UMC in the period December 2022 and January 2023. The study was approved by the Medical Ethics Committee and written consent was obtained from all participants.

### Participants

Participants consisted of surgical residents from the Netherlands, Greece, and Italy without any robotic experience. Only robotic novices, from all residency years, were included as surgical performance was based on previous robotic surgery experience and because stronger improvement is seen at the beginning of the learning curve, compared to improvement after training of robotic experts. A total of 20 participants were included and randomized in 2 groups with 10 participants in the robotic simulation-first group and 10 participants in the suture-pad-first group. Stereoptic abilities were assessed using a random dot TNO stereopsis test. Participants were excluded if they had no 3D vision (< 240 s of arc). Participants’ demographics, including baseline experience, and reported side effects and preferences were collected with a survey (see Appendix 1).

### Intervention

Participants received 15 min of instruction videos per e-mail and an oral instruction on-site prior to the experiment. During the training sessions, no instructions were provided. Participants used three robotic arms with the same set of two instruments, i.e., two needle drivers, and one 30-degree scope for all exercises. Artificial organ models were obtained from Heidelberg, i.e., bile duct [15], and Limbs and Things, i.e., long double-layer large bowel. The da Vinci X and Xi systems and the SimNow simulators were provided by Intuitive. The suture-pad training and robotic suturing exam were performed with monofilament tapered sutures: 20 cm Surgipro 4-0 CV-23 (B Braun, Melsungen, Germany).

**Fig. 2:**
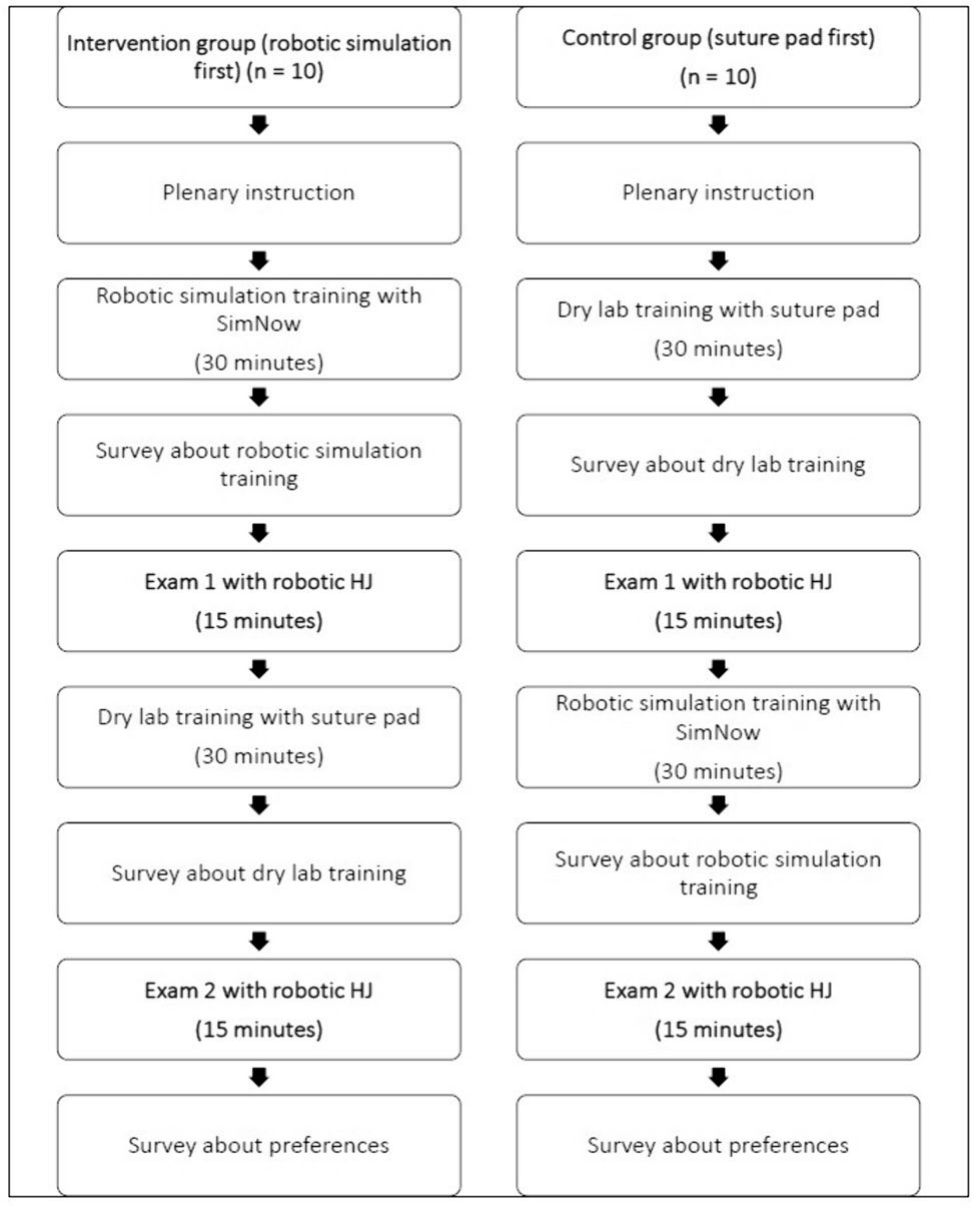
Flowchart of the training curricula of both groups

Ergonomic conditions were adjusted in advance of all exercises for each participant. The intervention group started with robotic simulation training, while the control group started with suture-pad training. The suture-pad training consisted of 30 minutes practice of interrupted transcutaneous suturing. The robotic simulation training consisted of repeatedly practicing exercise ‘Running suture’ for 15 minutes, followed by repeatedly practicing exercise ‘Knot tying’ for 15 minutes, aiming to practice handling of the needle driver and knot tying, respectively. The robotic simulation training included two different exercises as the SimNow software does not provide an exercise with complete suture repetitions, conform the suture-pad exercise. After the first training session, a 15-minute-robotic suturing exam was conducted in which the participants performed a HJ in biotissue, as far as possible. The participants had to stop the exam after 15 minutes. After the first exam, the groups switched training modality, followed by a second robotic suturing exam (the primary endpoint). The training curricula of both groups are presented in a flowchart in Fig. 2. A pre-test HJ was not performed, to adhere to the stepwise approach of the LAELAPS-3 program [16], and the HJ itself might impact the learning, resulting in biased and diluted effect sizes.

### Performance assessment

**Fig. 3:**
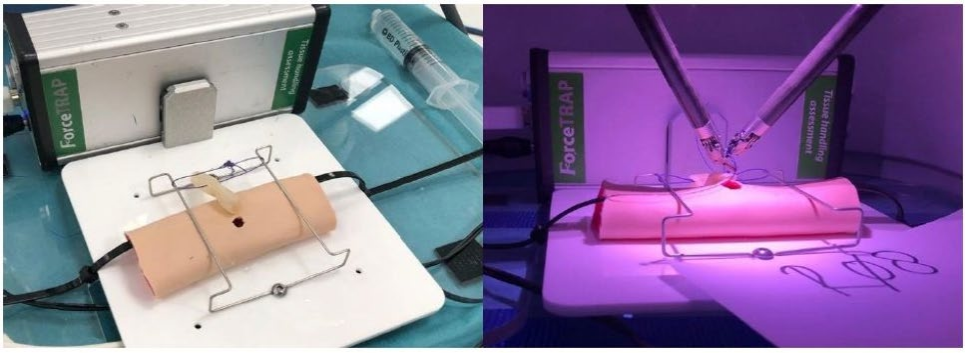
Robotic suturing exam (hepaticojejunostomy in biotissue) with objective force measurements

All videos were anonymously and separately presented to two blinded trained raters. Birkmeyer et al*.* validated the objective structured assessment of technical skills (OSATS) score to assess skills in laparoscopic surgery [17]. In the present study, surgical performance was graded using the modified OSATS score for RPD, as reported by Hogg et al. [18] (see Table 1). All exams were recorded and force measurements were made. The da Vinci video imaging was directly recorded, using Open Broadcast Software (OBS) Studio version 28.1.2, and backup videos were made with webcams connected to the ForceSense system (Medishield, Delft, the Netherlands). The force measurements were made with the ForceSense system (see Fig. 3).

**Table 1:** Modified OSATS grading as reported by Hogg et al*. *[17, 18, 37]

Rating and interpretation	
1	Deficient/traumatic
2	Lacking/lacks finesse
3	Average
4	Skilled
5	Master/flawless

Grading aspects and elucidation	
Gentleness	Gentle tissue handling that does not result in injury
Time and motion	Economy of motion, maximum efficiency
Instrument handling	Fluid use of instruments without awkwardness
Flow of operation	Smooth transitions from one part of the operation to another
Tissue exposure	Retraction that allows for good visualization and proper tissue alignment
Overall technical sdkill	Overall assessment of technical skill

### Randomization

Prior to the experiment, the participants were randomly assigned to the intervention group (robotic simulation-first) or control group (suture-pad-first) with IBM SPSS Statistics for MacOS version 29 (IBM Corp, Armonk, New York) by the study coordinator. Participants were unaware of the randomization sequence. The data were anonymized by using a randomly generated four-digit code. Only the principal investigator and study coordinators had access to the participants’ decoded data.

### Outcomes

The primary outcome was surgical performance after the second training, according to the modified OSATS score (see Table 1). The OSATS score is the mean score of the exam for all elements, scored per rater. Because of expected interrater variability, the primary outcome was defined as the combined OSATS score (the mean score of both raters combined, i.e., the cumulation of both OSATS scores divided by the number of raters). The secondary outcomes were force measurements at the second exam. The ForceSense system measured the exerted force on tissue per repetition (20 ms). The primary force analysis calculated the median force per group, based on the average force per exam within that group. The subsequent sub-analysis calculated the mean force per group, based on all repetitions of each exam.

The average force is the mean of all repetitions, including when Newton forces were zero. The non-zero force is the mean of repetitions only when pressure is exerted. Maximum force is the highest force measured in one repetition (20 ms), whereas Maximum impulse is the highest force calculated over 1 s (1000 ms). These forces are based on the average of all repetitions per exam. Forces on tissue are associated with tissue injury, with a benchmark of 4 Newtons [19, 20]. For an accurate prediction of potential tissue damage, forces of all repetitions were included and analyzed. Other outcomes were participants’ demographics (age, sex, level of residency, baseline experience, hand dominance, vision correction, and learning style) and experience and ergonomics with both training modalities. Previous experience with the da Vinci X/Xi system included general robotic experience in training or clinical setting. A qualitative analysis was performed to assess the participants’ opinion on training capacity and experienced side effects per training modality on a scale from 1 to 5 (5 = ‘best’ or ‘heaviest,’ respectively). Also, the participants’ preference on robotic training modality and sequence was reported.

### Sample size

No literature existed for both OSATS score and force measurements with robotic suturing training in surgical residents nor surgeons. Therefore, the sample size calculation was based on previous studies that used OSATS score only. These studies included up to 19 surgical residents [6, 8, 11, 13]. A sample size of 16 participants will have 80% power to detect an estimated effect size of 0.75 using a paired T-test with a 5% two-sided significance level. With a fall-out of around 20%, we expected to require 10 novices per group.

### Statistical methods

The OSATS score and force measurements of the second exam (i.e., after both trainings) were compared between the groups. Second, the OSATS score and force measurements of the first exam were compared. Potential trends in force on tissue per exam were analyzed using the non-parametric Spearman’s rho correlation test. The baseline experience, the consecutive performance speed, and learning curve during the exercises of both groups were expected to be equally distributed by computerized randomization. Primary analysis of the force measurements calculated the median force per group, using only one average force per exam. To visualize the occurrence of potential tissue damage, an additional sub-analysis was performed to calculate the mean force per group, including all repetitions (per 20 ms) of each exam. Finally, the correlation between the OSATS score and the force measurements was analyzed with a paired Spearman’s rho test. Data were analyzed using IBM SPSS Statistics for MacOS. After univariate analysis, sensitivity analysis was performed with select cases to adjust for potential baseline imbalances and an additional multivariate analysis to assess the impact of the independent variables on surgical performance. A qualitative analysis was performed to assess the participants’ overall experience with both training modalities. Normally distributed continuous data, including the OSATS score, were analyzed with an unpaired T-test, and presented as means and standard deviations (SDs). Non-normally distributed continuous data, including the force measurements, were analyzed with a non-parametric Mann–Whitney U test, and presented as medians and interquartile ranges [IQRs]. Categorical data, including the baseline demographics and qualitative analysis, were analyzed with the Chi-square test, and presented as frequencies and percentages. Likert-scale ordinal data were presented in means and standard deviations to enhance interpretation of the effect size. A two-tailed p-value of less than 0.05 was considered statistically significant.

## Results

### Participants

In December 2022 and January 2023, 20 robotic novices performed 40 HJs, i.e.*,* two exams per participant (see Fig. 1). Data of one HJ were partially lost due to a technical issue and were not repeated because of potential learning curve interference.

### Baseline demographics

In total, 20 robotic novices from 3 countries (Greece, Italy, the Netherlands) participated. The intervention group included 10 participants, who started with robotic simulation, followed by suture-pad training. The control group included 10 participants, who started with suture-pad, followed by robotic simulation training. Baseline characteristics between both groups were comparable (see Table 2). The mean age of participants was 31 years (± 2.5) without differences between the groups (31 ± 2.6 vs 31 ± 2.6; *p* = 0.971). In total, 13/20 participants were female, without differences between the groups (5/10 female vs 8/10 female; *p* = 0.350). The median level of surgical residency was the first year, and similar between both groups (1.0 [0.8–3.5] vs 2.0 [1.0–5.0]; *p* = 0.849). Three participants were surgical residents not in training. Baseline experience with open, laparoscopic, and robot-assisted suturing was similar between the groups. None of the participants had experience with robotic suturing. The only experience with robotic surgery reported consisted of changing the instruments as an assistant during robotic surgery. More detailed baseline demographics are presented in Supplementary Table 1.

**Table 2:** Baseline demographics of participants, intervention group (robotic simulation-first), and control group (suture-pad-first)

Participant characteristics	Total (*n* = 20)	Intervention group: first robotic simulation, second suture-pads (*n* = 10)	Control group: first suture-pads, second robotic simulation (*n* = 10)	*P* value
Age (years)	31 ± 2.5	31 ± 2.6	31 ± 2.6	0.971
Male (%)	7 (35%)	5 (50%)	2 (20%)	0.350
Level of residency (years)	1.0 [1.0–4.8]	1.0 [0.8–3.5]	2.0 [1.0–5.0]	0.849
No robotic sutures	20 (100%)	10 (100%)	10 (100%)	1.000
Right-hand dominance*	17 (85%)	9 (90%)	8 (80%)	1.000
Vision correction	11 (55%)	5 (50%)	6 (60%)	1.000
Experience with da Vinci	7 (35%)	3 (30%)	4 (40%)	1.000

Values are mean ± SD, median [quartile 1 to quartile 3], or *n* (percentage)

*None of the participants were ambidextrous

### Primary outcome

Due to expected significant interrater variety in OSATS score of the first exam (Kappa value = 0.20) and the second exam (Kappa value = 0.16) (see Supplementary Table 2), data analysis was performed with the combined OSATS score and the OSATS score per rater.

#### OSATS after the second training

In the second exam, the mean combined OSATS score was significantly higher in the robotic simulation-first group (3.3 ± 0.9 vs 2.5 ± 0.8, respectively; *p* = 0.049) (see Table 3). No individual OSATS elements were reported to be significantly different between groups by both raters. More detailed OSATS outcomes of the second exam are presented in Supplementary Table 3.

**Table 3:** Surgical performance in the second exam according to the Objective Structured Assessment of Technical Skill (OSATS) score

Mean OSATS score	Total (*n* = 20)	Intervention group: first robotic simulation, second suture-pads (*n* = 10)	Control group: first suture-pads, second robotic simulation (*n* = 10)	*P* value
OSATS 2—R1	2.8 ± 0.9	3.1 ± 0.9	2.5 ± 0.8	0.161
OSATS 2—R2	3.0 ± 1.0	3.5 ± 1.0	2.6 ± 0.8	0.037
Combined OSATS 2	2.9 ± 0.9	3.3 ± 0.9	2.5 ± 0.8	0.049

R1 is rater 1, R2 is rater 2. Values are mean ± SD

### Secondary outcomes

In the second exam after both trainings, no significant difference was found in average force, non-zero force, maximum force, and maximum impulse between groups (see Supplementary Table 4). One second exam in the suture-pad-first group only included force measurements of 3 min and 48 s, due to lost data. This incomplete exam was excluded from the sub-analysis. The sub-analysis included all repetitions to calculate the mean force per group.

#### Force after the second training

**Fig. 4:**
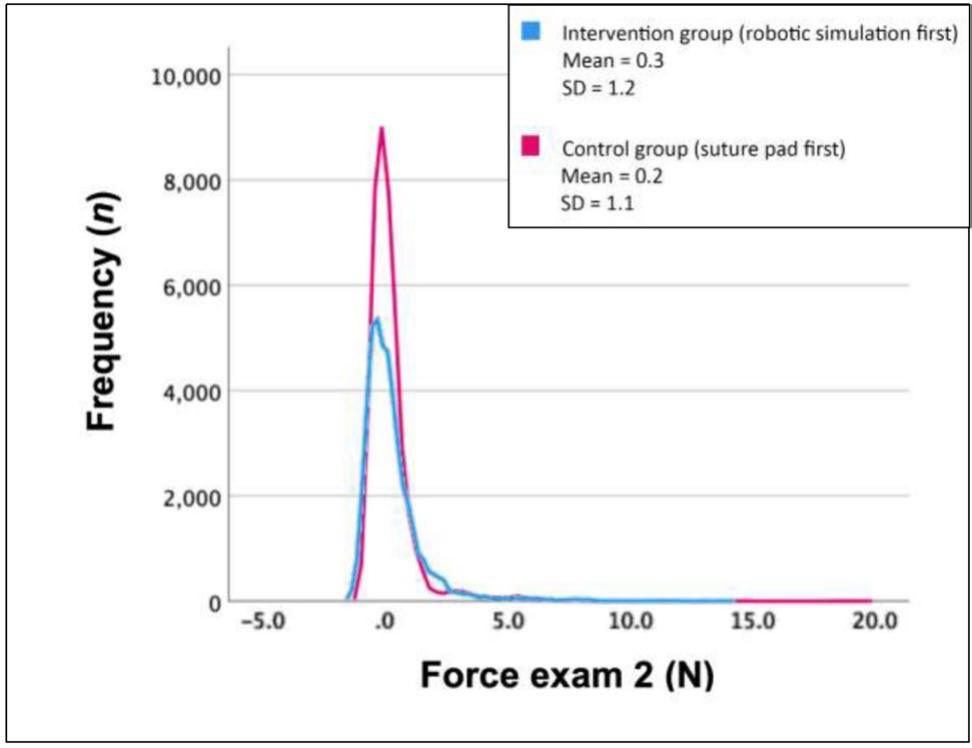
The number of repetitions that was measured per force value during the second exam in the robotic simulation-first group (*n* = 10) and suture-pad-first group (*n* = 9). Frequency (y-axis) presents the number of repetitions that were measured per force, expressed in *n*. Force (x-axis) presents the force on tissue in exam 2, expressed in Newton. One exam in the suture-pad-first group is excluded, due to incomplete data

In the second exam, the robotic simulation-first group exceeded the 4 Newton threshold (i.e., threshold for potential tissue damage) more frequently (see Supplementary Fig. 1). However, the suture-pad-first group exerted the highest force with 151.9 Newton, compared to 20.1 Newton in the robotic simulation-first group (see Fig. 4). In both groups, the average force significantly increased (*p* < 0.001 and *p* < 0.001) (see Supplementary Fig. 1).

### Effect baseline demographics on primary outcome

None of the baseline demographics was associated with the combined OSATS score for the second exam. More details are presented in Supplementary Table 5. In the sensitivity analysis, excluding participants with experience as assistant or experience with the da Vinci system in general, no difference was seen between groups in combined OSATS score for the second exam (2.9 ± 0.8 vs 2.6 ± 0.6; *p* = 0.463) (see Supplementary Table 6). Multivariate analysis did not show a significant effect of the two variables on combined OSATS score for the second exam (*p* = 0.539 and *p* = 0.254, respectively) (see Supplementary Table 7).

### Survey among participants

Suture-pad training was reported to be more realistic compared to robotic simulation training (4 [4] vs 2 [2, 3], respectively; *p* < 0.001). Didactic value was scored higher for suture-pad training than robotic simulation training (5 [4, 5] vs 4 [3, 4], respectively; *p* = 0.007). Also, feasibility for robotic suturing training was rated higher for suture-pad training than robotic simulation training (5 [4, 5] vs 4 [3, 4], respectively; *p* < 0.001). An experience of disorientation was reported to be more severe with robotic simulation compared to suture-pad (2 [1, 2] vs 1 [1, 2]; *p* = 0.008). Other experienced side effects were similar between both groups. More detailed outcomes on the participants’ experience with both training modalities are presented in Supplementary Table 8. Seventeen out of 20 (85%) participants recommended to include robotic simulation training with the SimNow simulator in the robotic training curriculum. All 20 participants agreed on including suture-pad training into the curriculum. Fourteen out of 20 (70%) participants preferred to start with robotic simulation training followed by suture-pad, whereas 6/20 (30%) preferred to start with suture-pad training followed by robotic simulation. No difference was found between groups in opinion on including robotic simulation and suture-pad for robotic suturing training (*p* = 1.000 and *p* = 1.000, respectively) nor preferences for curriculum (*p* = 0.628). More detailed outcomes on training preferences are presented in Supplementary Table 9.

### Validation of force measurements

Gentleness, *i.e.*, the OSATS grading aspect for tissue handling, was compared with force on tissue. This analysis showed a consistent negative correlation between the mean gentleness score and force measurements of both exams (see Supplementary Tables 10 and 11).

### Results of first exam

#### OSATS

In the first exam, the mean combined OSATS score was similar between groups (2.4 ± 0.8 vs 2.4 ± 0.7; p = 0.791). More detailed OSATS outcomes of the first exam are presented in Supplementary Table 12.

#### Force

No significant difference was found in average force, non-zero force, maximum force, or maximum impulse between groups in the first exam (see Supplementary Table 13). In the first exam, force frequently exceeded the 4 Newton threshold for tissue damage in both groups (see Supplementary Fig. 2). In both groups, the mean force significantly decreased over time (*p* < 0.001 and *p* < 0.001) and the highest force was 10.2 Newton (see Supplementary Fig. 3).

#### Predictors for OSATS

Experience as assistant in robotic surgery (*p* = 0.025) and experience with the da Vinci system in general (*p* = 0.006) were correlated with an increased combined OSATS score for the first exam. Sensitivity analysis showed an impact as no difference was seen between groups in combined OSATS score for the first exam (2.0 ± 0.4 vs 2.2 ± 0.5; *p* = 0.885) (see Supplementary Table 6). Multivariate analysis did not show a significant effect of the two variables on combined OSATS score for the first exam (*p* = 0.800 and *p* = 0.127, respectively) (see Supplementary Table 7). More details on the correlation between baseline demographics and the combined OSATS score of the first exam are presented in Supplementary Table 14.

## Discussion

This experimental, randomized controlled crossover trial for robotic HJ is the first study to compare surgical performance subjectively and objectively with the OSATS score and force measurements in robotic novices who started training with either robotic simulation or suture-pads. This study is also the first to compare the effect of the new SimNow with suture-pad training on robotic suturing by novices. We found improved surgical performance in novices who had started with robotic simulation training followed by suture-pad training, with a 32% higher OSATS score as compared to starting with suture-pad followed by robotic simulation.

Since this is the first study to compare sequences of surgical training in robotic suturing, we cannot compare our results. Radi et al*.* included 41 surgical residents who performed 33 robotic simulation drills with the SimNow, among which were the ‘Running suture’ and ‘Knot tying’ exercises. All participants conducted a pre- and post-test, including robotic simulation and suture-pad tasks, rated according to the OSATS score. Surgical performance significantly improved after completing the robotic simulation training [21]. Tellez et al*.* also reported a significant improvement in both robotic simulation and inanimate surgical performance, according to the OSATS score, after training surgical residents with the SimNow’s 33 robotic simulation drills [22]. However, these studies did not compare the SimNow with suture-pad training.

One of the greatest challenges of robot-assisted surgery is tissue handling given the absence of haptic feedback. Excessive forces may lead to irreversible tissue damage [20]. Hence, force measurements assess tissue handling and predict subsequent occurrence of tissue damage. Maximum force and maximum impulse may predict potential tissue damage as validated by a recent study [19]. The present study demonstrated a correlation between the subjective and objective assessment of tissue handling with the OSATS grading aspect ‘gentleness’ and the force measurements. Interestingly, in the second exam, the robotic simulation-first group exceeded the 4 Newton threshold more often and therefore potentially caused tissue damage more frequently than the suture-pad-first group. This finding contradicts the positive association between OSATS ‘gentleness’ and force measurement. Tissue damage cannot be predicted by median force only as a single exceedance of the 4 Newton threshold is sufficient to potentially cause tissue damage.

Robotic simulation training has multiple benefits over dry-lab training. First, robotic simulation is more accessible with less set-up time with only a surgeon’s console and the SimNow backpack, compared to dry-lab training with the complete da Vinci system, instruments, and materials. Besides, with dry-lab training, accurate force feedback can only be given in the presence of the ForceSense system [23]. Second, robotic simulation is less expensive than dry-lab training [12]. Dry-lab training requires the complete da Vinci surgical system, annual maintenance, disposable and reusable instruments, artificial organ models, and a ForceSense system [24, 25]. Robotic simulation training only requires a single surgeon’s console and the SimNow platform, which is often included in the purchase of a da Vinci Xi system [26]. Dry-lab training includes realistic tissue handling and instrument interaction, but robotic simulation software is rapidly developing. Besides, the new SimNow platform includes advanced exercises for practice of robotic skills and complete guided and non-guided procedures of multiple surgical disciplines (e.g., cholecystectomy, hemicolectomy, and inguinal hernia repair). The software also offers various curricula and objective assessment of personal skills development [5].

Although the participants preferred suture-pad training over robotic simulation training, the qualitative analysis showed that the majority of the participants agreed on including the SimNow in a robotic suturing curriculum. Despite suture-pad training being rated significantly better, the SimNow was considered sufficiently didactic and feasible for robotic suturing training in novices. However, it cannot replace suture-pad training. Turbati et al*.* investigated five drills of the SimNow for robotic training in surgical residents and provided the first qualitative analysis on its training capacity. This study validated their curriculum for robotic training in surgical residents and described the SimNow’s ability to distinguish between robotic novices and competent or expert surgeons [27]. However, Turbati et al*.* did not investigate suturing drills like the exercises ‘Running suture’ and ‘Knot tying’ that were included in this study.

Pancreatoduodenectomy (PD) is one of the most complex abdominal surgical procedures. Robot-assisted pancreatoduodenectomy (RAPD) rapidly developed over the past few decades, aiming to improve intra- and postoperative patient outcomes as compared to open and laparoscopic PD [28, 29]. In the Netherlands, the LAELAPS-3 training program resulted in a nationwide implementation of RAPD [30]. Thereafter, this evolved into the European LEARNBOT program by the European consortium on Minimally Invasive Pancreatic Surgery (E-MIPS). Both training programs included robotic simulation and suture-pad exercises, the latter including practice of the reconstruction phase of RAPD, among which was an HJ in artificial organ models [15, 16, 30]. Surgical performance in both training programs was graded using the OSATS score as this tool is not only validated for the assessment of the learning curve but can also be used for quality control by predicting the risk of postoperative pancreatic fistula and bile leakage after RAPD [30–33]. The difference of 0.8 in final OSATS score as reported in the present study could thus have a clinical impact on the incidence of postoperative complications. Increased experience with RAPD is associated with improved intra- and postoperative patient outcomes, suggesting adequate training prior to live procedures to be beneficial for safe implementation of robotic surgery [34, 35]. Hence, the creation of an adequate robotic training curriculum is essential to optimize the learning curve. This study validated the stepwise approach according to the IDEAL framework, which is used in the completed Dutch LAELAPS-3 and the ongoing European LEARNBOT training programs for implementation of robotic pancreatoduodenectomy, including HJ in the reconstruction phase [30, 36]. These training programs start with theory, followed by robotic simulation training with the SimNow, dry-lab training, case observations, and finally a proctored case. This approach is also useful for proper implementation of a standardized training program during surgical residency or fellowship. The hypothesis is that initial robotic simulation exposure enables practice with the system and its features, resulting in enhanced performance during consecutive dry-lab training. With the opportunity to choose training sequence, the present study recommends a training program during residency or fellowship to start with robotic simulation followed by suture-pad rather than opposite sequence. In case of shortage in training capacity, practice with either modality could be sufficient as the first exam showed 0% difference in robotic suturing performance after single training with the SimNow versus single training with the suture-pad in terms of OSATS score and force on tissue.

The results of the present study should be interpreted in light of some limitations. First, the participants expressed a strong preference for suture-pad training. Although the surveys were completed directly after the training session (to preclude the exam’s performance to have an impact on the assessment of the training modalities), the qualitative analysis might have been affected by this confirmation bias. Second, the sensitivity analysis, excluding participants with some experience as assistant during robotic procedures or the da Vinci system in general, had an impact as it showed no significant difference in surgical performance according to the OSATS score between groups for both exams. Yet, we assume this finding is a matter of statistical power as the groups might have been too small after excluding participants with experience as assistant or experience with the da Vinci system in general (*n* = 7 in the robotic simulation-first group, *n* = 6 in the suture-pad-first group). Third, force measurements may be influenced by the progress of the anastomosis, which is not included in the surgical performance assessment. Fourth, the present study focused on final surgical performance, *i.e.,* after both training modalities. Despite the non-inferior surgical performance between the groups in the first exam, the study cannot draw evident conclusions if the more accessible robotic simulation training with the SimNow can replace the suture-pad training with more expensive training instruments in robotic novices. Future studies are needed to compare both sequences with only suture-pad training (suture-pad followed by suture-pad training). Strengths of this study included the two raters with both OSATS and objective force measurements in a randomized crossover study design including two types of training for robotic suturing.

In conclusion, surgical performance of robotic novices was significantly better after robotic simulation-first training followed by suture-pad training as compared to the opposite sequence. Participants considered robotic simulation training with the SimNow to be of added value for robotic suturing training, however not as a substitute for suture-pad training. An optimal robotic suturing curriculum should thus combine both robotic simulation and suture-pad suturing and it should start with robotic simulation.

## Supplementary Information

Below is the link to the electronic supplementary material.

Supplementary file1 (DOCX 3944 KB)
