# Supplementary material for: Robotic hepaticojejunostomy training in novices using robotic simulation and dry-lab suturing (ROSIM): randomized controlled crossover trial
Source: Surg Endosc. 2024 Jul 3;38(9):4906–15. doi: 10.1007/s00464-024-10914-8 (PMC11362386; doi:10.1007/s00464-024-10914-8)

**SUPPLEMENTS**

**Supplementary figure 1:** Detailed force measurements per repetition of the second robotic hepaticojejunostomy exam.

| 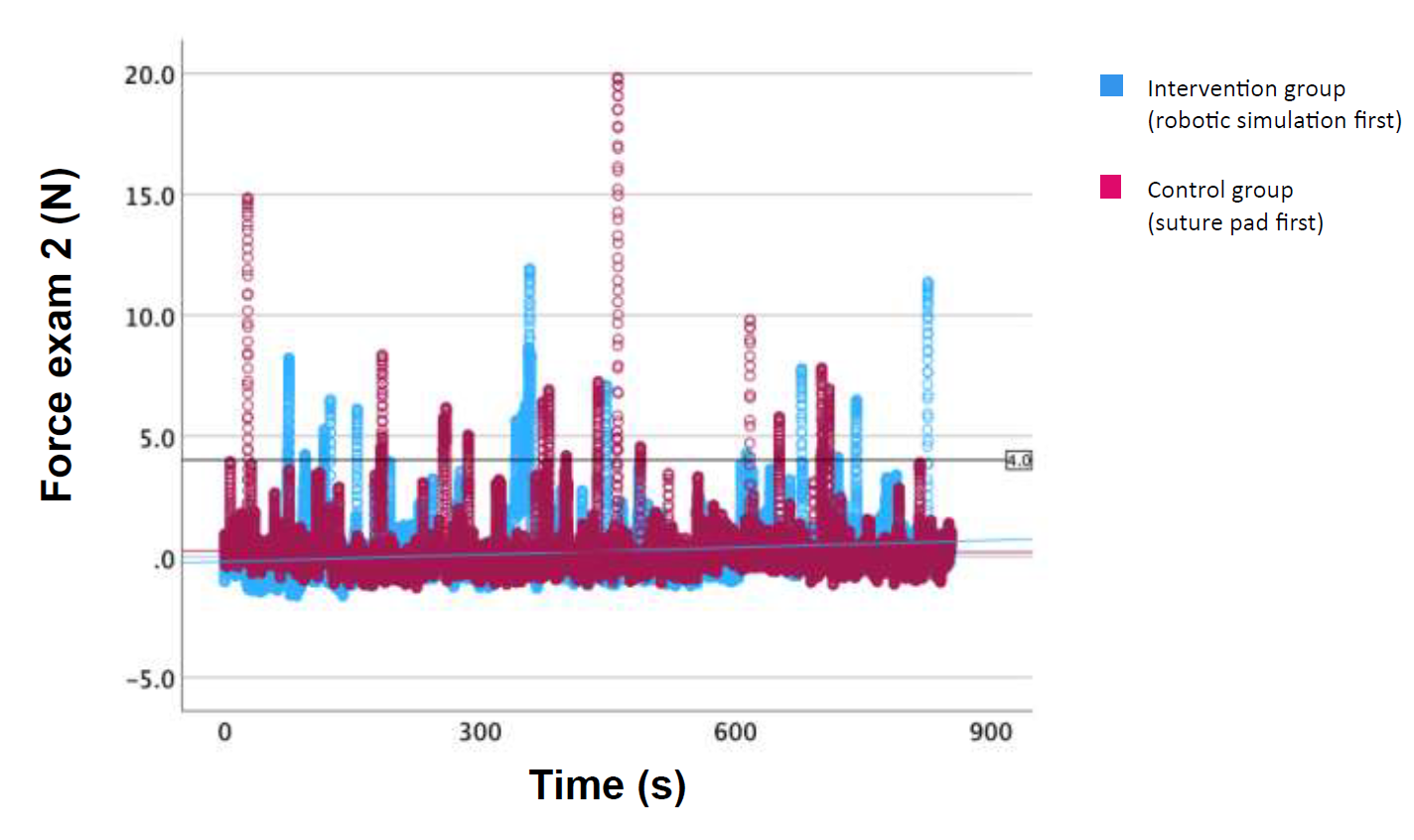 |
| --- |
| 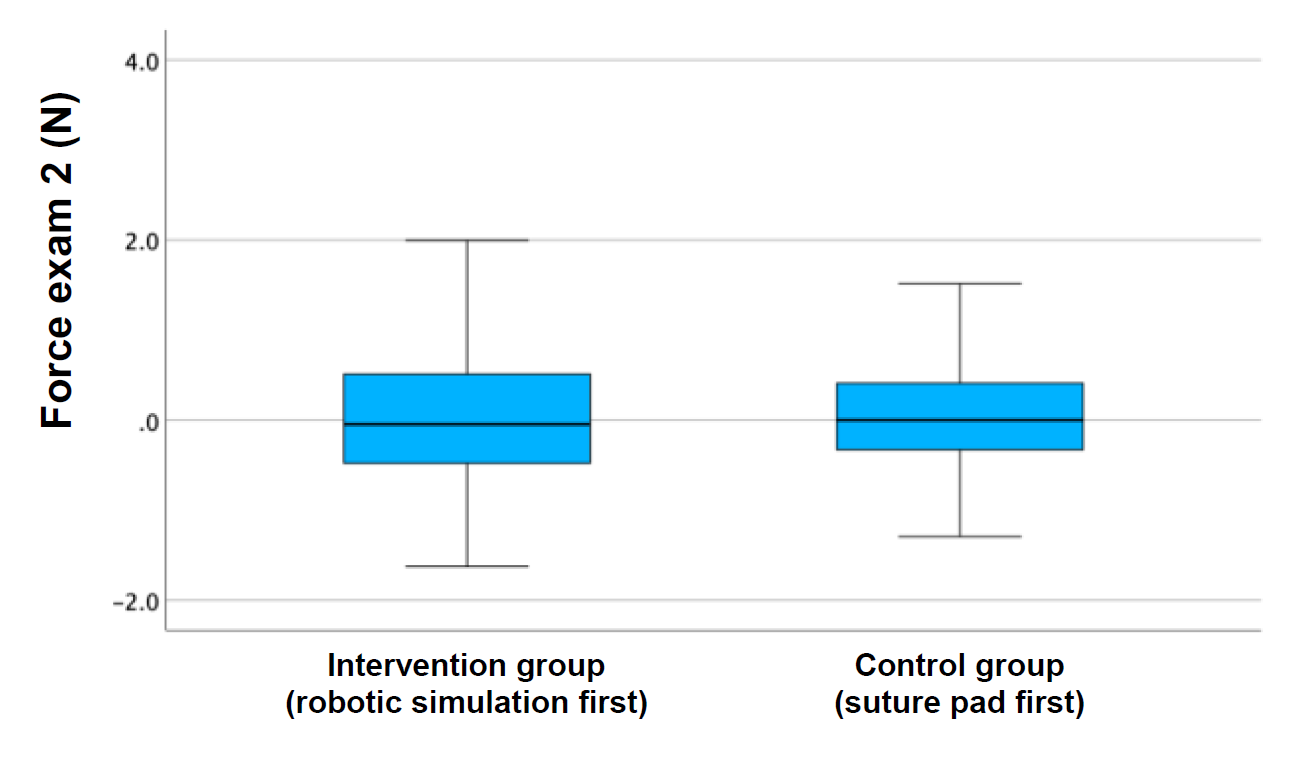 |

**^Legend supplementary figure 1:^** ^Force on tissue in the second exam of the intervention group (robotic simulation-first, n=10) and control group (suture-pad-first, n=10). Values are force (N) or time (s). The threshold for tissue damage is 4 Newtons. Outliers are excluded from the boxplot.^

**Supplementary figure 2:** Detailed force measurements per repetition of the first exam.

| 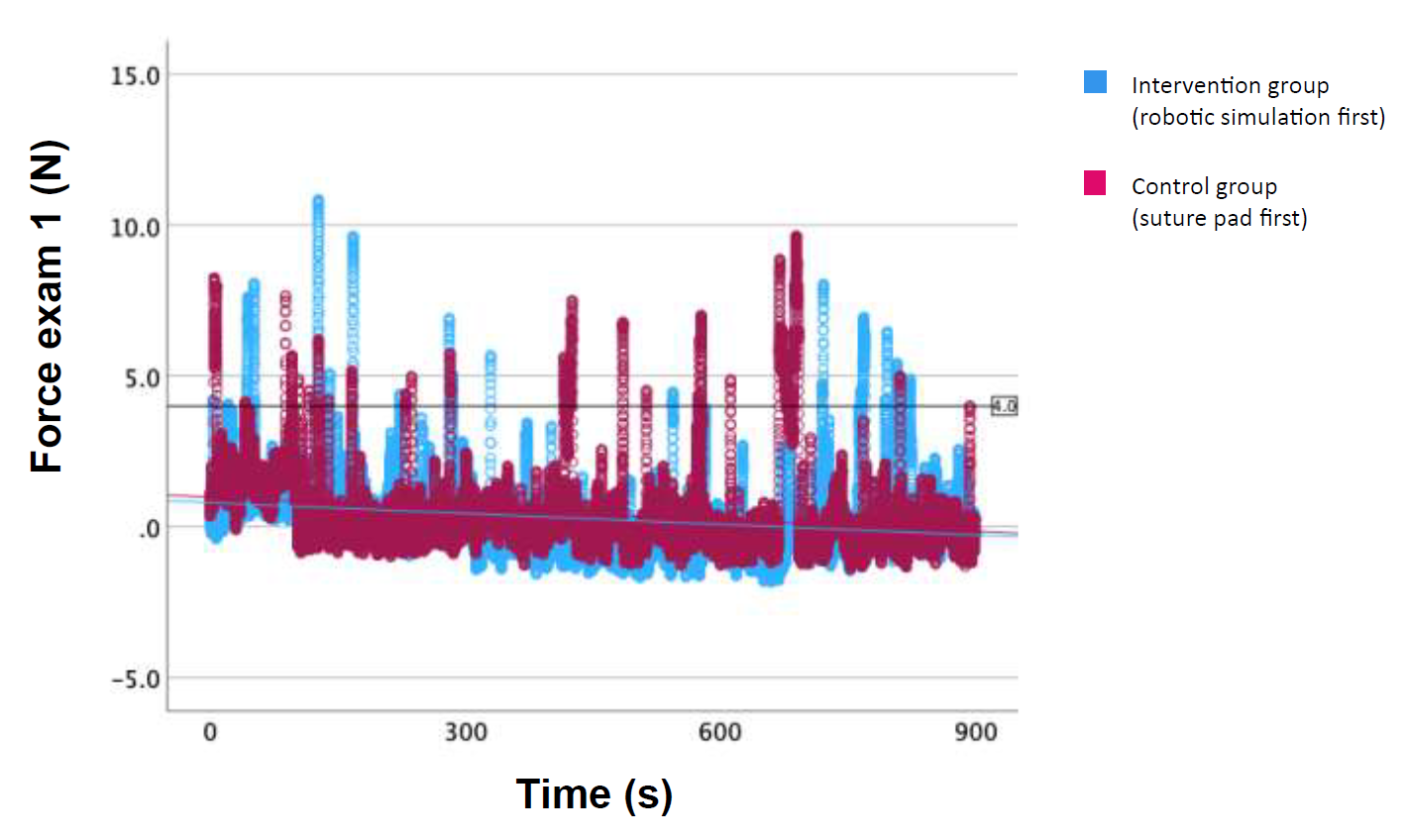 |
| --- |
| 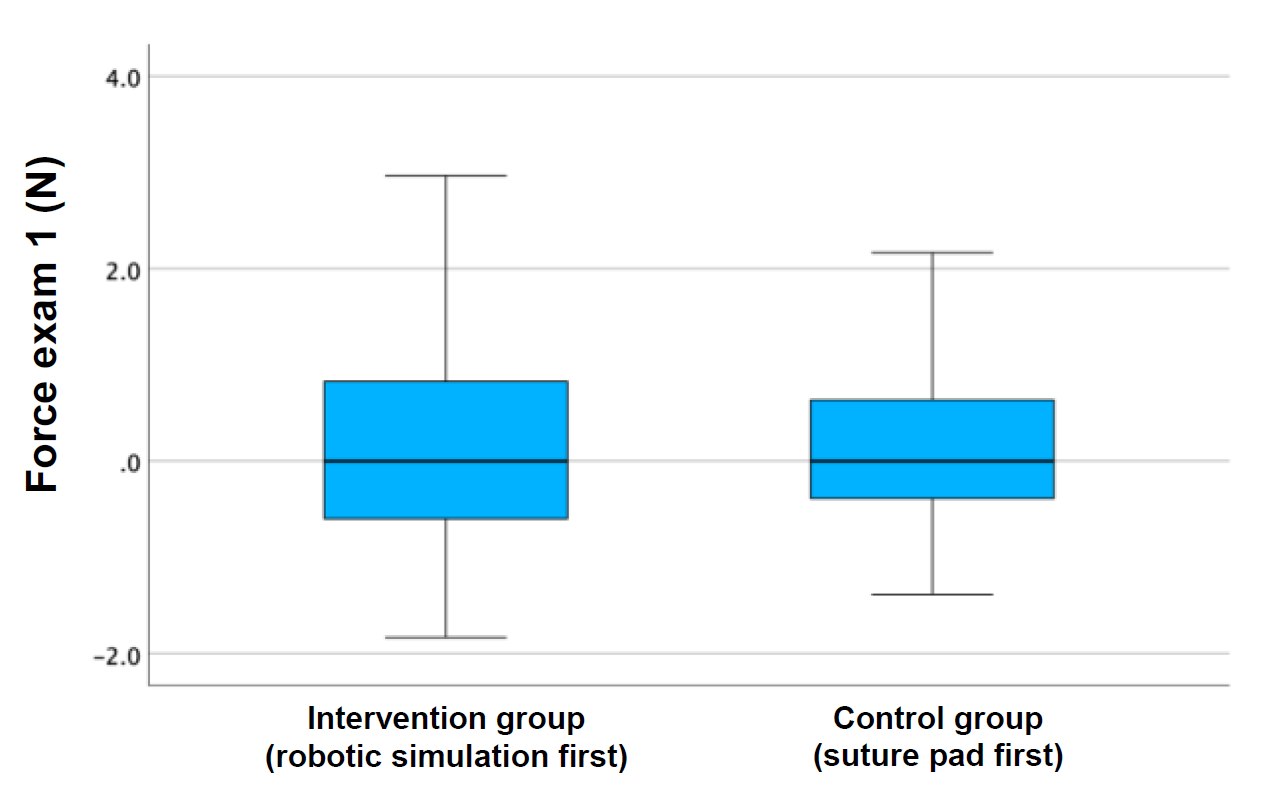 |

**^Legend supplementary figure 2:^** ^Force on tissue in the first exam of the intervention group (robotic simulation-first, n=10) and control group (suture-pad-first, n=10). Values are force (N) or time (s). The threshold for tissue damage is 4 Newtons. Outliers are excluded from the boxplot.^

**Supplementary figure 3:** The number of repetitions that was measured per force value during the first exam in the robotic simulation-first group (n=10) and suture-pad-first group (n=10).


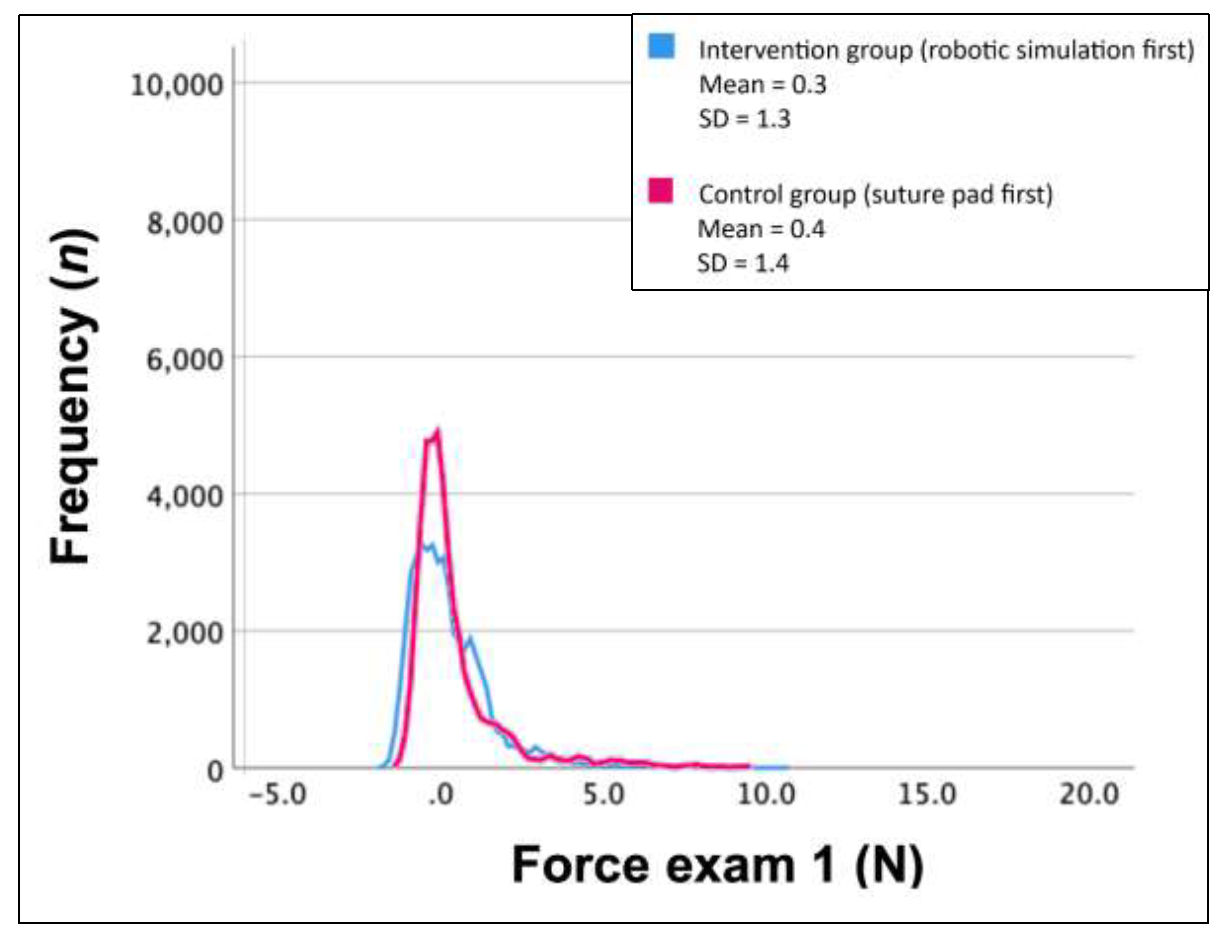
**^Legend supplementary figure 3:^** ^Frequency (y-axis) presents the number of repetitions that were measured per force, expressed in ­^*^n^*^. Force (x-axis) presents the force on tissue in exam 1, expressed in Newton.^

**Supplementary table 1:** Detailed baseline demographics.

| **Baseline demographics** | **Total**  **(n = 20)** | **Intervention group: first robotic simulation, second suture-pads**  **(n = 10)** | **Control group: first suture-pads, second robotic simulation**  **(n = 10)** | **P-value** |
| --- | --- | --- | --- | --- |
| Age (years) | 31 ± 2.5 | 31 ± 2.6 | 31 ± 2.6 | 0.971 |
| Female sex (%) | 13 (65%) | 5 (50%) | 8 (80%) | 0.350 |
| Level of residency | 1.0 [1.0–4.8] | 1.0 [0.8–3.5] | 2.0 [1.0–5.0] | 0.849 |
| - Not in training | 3 (15%) | 2 (20%) | 1 (10%) |  |
| - 1^st^ year | 8 (40%) | 4 (40%) | 4 (40%) |  |
| - 2^nd^ year | 1 (5%) | 1 (10%) | 0 (0%) |  |
| - 3^rd^ year | 2 (10%) | 1 (10%) | 1 (10%) |  |
| - 4^th^ year | 1 (5%) | 0 (0%) | 1 (10%) |  |
| - 5^th^ year | 3 (15%) | 1 (10%) | 2 (20%) |  |
| - 6^th^ year | 2 (10%) | 1 (10%) | 1 (10%) |  |
| No robotic procedures – Chief | 20 (100%) | 10 (100%) | 10 (100%) | 1.000 |
| Robotic procedures – Assistant |  |  |  | 0.153 |
| - 0 procedures | 11 (55%) | 6 (60%) | 5 (50%) |  |
| - 1-5 procedures | 6 (30%) | 4 (40%) | 2 (20%) |  |
| - >5 procedures | 3 (15%) | 0 (0%) | 3 (30%) |  |
| Hand dominance |  |  |  | 1.000 |
| - Right | 17 (85%) | 9 (90%) | 8 (80%) |  |
| - Left | 3 (15%) | 1 (10%) | 2 (20%) |  |
| Vision correction | 11 (55%) | 5 (50%) | 6 (60%) | 1.000 |
| Learning style |  |  |  | 1.000 |
| - Assimilator | 6 (30%) | 3 (30%) | 3 (30%) |  |
| - Accommodator | 14 (70%) | 7 (70%) | 7 (70%) |  |
| Experience with da Vinci | 7 (35%) | 3 (30%) | 4 (40%) | 1.000 |

**^Legend supplementary table 1:^** ^Values are mean ± SD, median [quartile 1 to quartile 3], or^ *^n^* ^(percentage).^

**Supplementary table 2:** Interrater reliability analysis.

| **Interrater reliability** | **Kappa value** |
| --- | --- |
| **OSATS 1** | **0.20** |
| OSATS 1 Gentleness | 0.47 |
| OSATS 1 Time and Motion | 0.15 |
| OSATS 1 Instrument Handling | 0.50 |
| OSATS 1 Tissue Exposure | 0.40 |
| OSATS 1 Flow of Operation | 0.41 |
| OSATS 1 Summary | 0.47 |
| OSATS 1 Cumulative | 0.20 |
| **OSATS 2** | **0.16** |
| OSATS 2 Gentleness | 0.38 |
| OSATS 2 Time and Motion | 0.38 |
| OSATS 2 Instrument Handling | 0.59 |
| OSATS 2 Tissue Exposure | 0.11 |
| OSATS 2 Flow of Operation | 0.37 |
| OSATS 2 Summary | 0.73 |
| OSATS 2 Cumulative | 0.16 |

**^Legend supplementary table 2:^** ^Interrater variety analyzed per element of the Objective Structured Assessment of Technical Skill (OSATS) score of the first and second exam. Values are Kappa value.^

**Supplementary table 3:** Detailed OSATS scores of the second exam.

| **Exam 2** | **Total**  **(n = 20)** | **Intervention group: first robotic simulation, second suture-pads**  **(n= 10)** | **Control group: first suture-pads, second robotic simulation**  **(n = 10)** | **P-value** |
| --- | --- | --- | --- | --- |
| OSATS Gentleness (R1) | 2.9 ± 0.8 | 3.1 ± 0.7 | 2.7 ± 0.8 | 0.297 |
| OSATS Gentleness (R2) | 3.0 ± 1.2 | 3.7 ± 1.0 | 2.3 ± 1.0 | 0.007 |
| OSATS Time and Motion (R1) | 2.7 ± 1.1 | 3.0 ± 1.2 | 2.4 ± 1.0 | 0.236 |
| OSATS Time and Motion (R2) | 3.0 ± 1.1 | 3.2 ± 1.0 | 2.7 ± 1.1 | 0.284 |
| OSATS Instrument Handling (R1) | 3.0 ± 1.0 | 3.4 ± 1.0 | 2.5 ± 0.9 | 0.048 |
| OSATS Instrument Handling (R2) | 3.1 ± 1.1 | 3.5 ± 1.1 | 2.6 ± 1.0 | 0.078 |
| OSATS Tissue Exposure (R1) | 2.8 ± 1.0 | 3.1 ± 1.0 | 2.4 ± 0.8 | 0.073 |
| OSATS Tissue Exposure (R2) | 3.0 ± 1.1 | 3.4 ± 1.2 | 2.6 ± 0.8 | 0.135 |
| OSATS Flow of Operation (R1) | 2.7 ± 1.0 | 2.9 ± 0.9 | 2.5 ± 1.1 | 0.407 |
| OSATS Flow of Operation (R2) | 3.1 ± 1.0 | 3.6 ± 1.0 | 2.6 ± 0.7 | 0.021 |
| OSATS Summary (R1) | 2.9 ± 1.0 | 3.2 ± 1.0 | 2.5 ± 0.9 | 0.142 |
| OSATS Summary (R2) | 3.1 ± 1.1 | 3.6 ± 1.1 | 2.6 ± 0.8 | 0.041 |
| OSATS Cumulative (R1) | 16.9 ± 5.4 | 18.7 ± 5.3 | 15.0 ± 5.0 | 0.161 |
| OSATS Cumulative (R2) | 18.2 ± 5.8 | 21.0 ± 5.7 | 15.4 ± 4.7 | 0.037 |
| **OSATS (R1)** | **2.8 ± 0.9** | **3.1 ± 0.9** | **2.5 ± 0.8** | **0.161** |
| **OSATS (R2)** | **3.0 ± 1.0** | **3.5 ± 1.0** | **2.6 ± 0.8** | **0.037** |
| **Combined OSATS** | **2.9 ± 0.9** | **3.3 ± 0.9** | **2.5 ± 0.8** | **0.049** |

**^Legend supplementary table 3:^** ^The Objective Structured Assessment of Technical Skill (OSATS) score of the second exam per element. The OSATS score per rater is the average of all elements combined. The OSATS summary score per rater reflects the overall technical skill. The combined OSATS score is the average of both raters combined. R1 is rater 1, R2 is rater 2.^

**Supplementary table 4:** Detailed force measurements of the second exam.

| **Exam 2** | **Total**  **(n = 20)** | **Intervention group: first robotic simulation, second suture-pads**  **(n = 10)** | **Control group: first suture-pads, second robotic simulation**  **(n = 10)** | **P-value** |
| --- | --- | --- | --- | --- |
| Average force | 0.3 [0.2–0.4] | 0.3 [0.3–0.3] | 0.3 [0.2–0.4] | 0.853 |
| Non-Zero force | 0.5 [0.4–0.6] | 0.4 [0.4–0.6] | 0.5 [0.4–0.7] | 0.529 |
| Maximum force | 4.6 [2.7–8.6] | 5.0 [3.2–8.0] | 3.8 [2.3–12.8] | 0.739 |
| Maximum impulse | 12.8 [8.0–21.5] | 15.4 [7.7–19.5] | 11.7 [8.8–40.5] | 0.971 |

**^Legend supplementary table 4:^** ^Force on tissue in the second exam, based on the average of all repetitions per exam (N). Values are median [quartile 1 to quartile 3] or p-value.^

**Supplementary table 5:** Details on the effect of baseline demographics on surgical performance in the second exam.

| **Baseline demographics** | **Combined OSATS 2** | |
| --- | --- | --- |
|  | **Correlation Coefficient** | **P-value** |
| Age | 0.004 | 0.987 |
| Gender | 0.091 | 0.702 |
| Level of residency | 0.216 | 0.360 |
| Open sutures | 0.354 | 0.126 |
| Laparoscopic sutures | 0.160 | 0.501 |
| Laparoscopy training - Simulation | 0.239 | 0.310 |
| Laparoscopy training - Dry-lab | 0.215 | 0.362 |
| Laparoscopy training - Wet lab | -0.091 | 0.702 |
| Laparoscopic procedures - Chief operator | 0.262 | 0.265 |
| Laparoscopic procedures - Assistant | 0.109 | 0.647 |
| Robot training - Simulation | -0.140 | 0.557 |
| Robotic procedures - Assistant | 0.165 | 0.488 |
| Hand dominance | 0.073 | 0.760 |
| Vision correction | 0.201 | 0.396 |
| Learning style | 0.019 | 0.937 |
| Experience with da Vinci | -0.273 | 0.243 |

**^Legend supplementary table 5:^** ^Univariate analysis of the baseline demographics and the combined Objective Structured Assessment of Technical Skills (OSATS) score in the second exam. Values are Correlation Coefficient or p-value.^

**Supplementary table 6:** Sensitivity analysis.

| **Sensitivity analysis** | **Intervention group: first robotic simulation, second suture-pads**  **(n = 7)** | **Control group: first suture-pads, second robotic simulation**  **(n = 6)** | **P-value** |
| --- | --- | --- | --- |
| Combined OSATS 1 | 2.0 ± 0.4 | 2.2 ± 0.5 | 0.885 |
| Combined OSATS 2 | 2.9 ± 0.8 | 2.6 ± 0.6 | 0.463 |

**^Legend supplementary table 6:^** ^Sensitivity analysis with select cases for surgical performance in the first and second exam, according to the Objective Structured Assessment of Technical Skills (OSATS) score. Values are mean ± SD.^

**Supplementary table 7:** Multivariate analysis.

| **Multivariate analysis** | **Combined OSATS 1** | **Combined OSATS 2** |
| --- | --- | --- |
| Robotic procedures –  Assistant | 0.440 | 0.440 |
| Experience with da Vinci | 0.907 | 0.907 |

**^Legend supplementary table 7:^** ^Multivariate analysis for surgical performance in the first and second exam, according to the Objective Structured Assessment of Technical Skills (OSATS) score. Values are p-value.^

**Supplementary table 8:** Qualitative analysis of the participants’ experience.

| **Qualitative analysis** | **Robotic simulation training** | **Suture-pad training** | **P-value** |
| --- | --- | --- | --- |
| **Training capacity (5 = best)** |  |  |  |
| - Realistic | 2 [2–3] | 4 [4–4] | **<0.001** |
| - Didactic | 4 [3–4] | 5 [4–5] | **0.007** |
| - Feasible for training | 4 [3–4] | 5 [4–5] | **<0.001** |
| **Side-effects (5 = heaviest)** |  |  |  |
| - Eye strain | 1 [1–2] | 1 [1–2] | 1.000 |
| - Headache | 1 [1–2] | 1 [1–1] | 0.687 |
| - Dizziness | 1 [1–2] | 1 [1–1] | 0.125 |
| - Disorientation | 2 [1–2] | 1 [1–2] | **0.008** |
| - Physical discomfort | 2 [1–2] | 2 [1–2] | 1.000 |
| - Poor visualization | 1 [1–3] | 1 [1–2] | 0.219 |

**^Legend supplementary table 8:^** ^Training capacity of and side-effects with robotic simulation (SimNow) and suture-pad training. Outcomes are based on a scale from 1 to 5, whereas 5 is best for training capacity and 5 is heaviest for experienced side-effects. Values are median [quartile 1 to quartile 3] or p-value.^

**Supplementary table 9:** Qualitative analysis of the participants’ preferences on robotic training curriculum.

| **Qualitative analysis** | **Total**  **(n = 20)** |
| --- | --- |
| **Include robotic simulation*** |  |
| - Yes | 17 (85%) |
| - No | 3 (15%) |
| **Include suture-pad*** |  |
| - Yes | 20 (100%) |
| **Start curriculum with*** |  |
| - Robotic simulation | 14 (70%) |
| - Dry-lab | 6 (30%) |

**^Legend supplementary table 9:^** ^Qualitative analysis of a robotic training curriculum with robotic simluation (SimNow) and suture-pad training. Values are^ *^n^* ^(percentage). *No statistically significant difference was found between the intervention (robotic simulation-first) and control (suture-pad-first) group.^

**Supplementary table 10:** Correlation OSATS score and force in the first exam.

| Exam 1 | OSATS Gentleness (R1) | | OSATS Gentleness (R2) | |
| --- | --- | --- | --- | --- |
|  | Correlation Coefficient | P-value | Correlation Coefficient | P-value |
| Average force | -0.301 | 0.197 | -0.600 | **0.005** |
| Non-zero force | -0.344 | 0.137 | -0.573 | **0.008** |
| Maximum force | -0.219 | 0.353 | -0.450 | **0.046** |
| Maximum impulse | -0.270 | 0.249 | -0.649 | **0.002** |

**^Legend supplementary table 10:^** ^Correlation between mean gentleness,^ *^i.e.^*^, the Objective Structured Assessment of Technical Skills (OSATS) grading aspect for tissue handling, and force measurements of the first exam. R1 is rater 1, R2 is rater 2. Values are Correlation Coefficient or p-value.^

**Supplementary table 11:** Correlation OSATS score and force in the second exam.

| Exam 2 | OSATS Gentleness (R1) | | OSATS Gentleness (R2) | |
| --- | --- | --- | --- | --- |
|  | Correlation Coefficient | P-value | Correlation Coefficient | P-value |
| Average force | -0.412 | 0.071 | -0.283 | 0.227 |
| Non-zero force | -0.463 | **0.040** | -0.350 | 0.130 |
| Maximum force | -0.330 | 0.155 | -0.238 | 0.313 |
| Maximum impulse | -0.403 | 0.078 | -0.148 | 0.535 |

**^Legend supplementary table 11:^** ^Correlation between mean gentleness,^ *^i.e.^*^, the Objective Structured Assessment of Technical Skills (OSATS) grading aspect for tissue handling, and force measurements of the second exam. R1 is rater 1, R2 is rater 2. Values are Correlation Coefficient or p-value.^

**Supplementary table 12:** Detailed OSATS scores of the first exam.

| Exam 1 | Total  (n = 20) | Intervention group: first robotic simulation, second suture-pads  (n = 10) | Control group: first suture-pads, second robotic simulation  (n = 10) | P-value |
| --- | --- | --- | --- | --- |
| OSATS Gentleness (R1) | 2.4 ± 0.8 | 2.3 ± 0.8 | 2.4 ± 0.7 | 0.590 |
| OSATS Gentleness (R2) | 2.5 ± 0.9 | 2.6 ± 1.0 | 2.3 ± 0.8 | 0.548 |
| OSATS Time and Motion (R1) | 2.4 ± 1.2 | 2.6 ± 1.4 | 2.1 ± 1.0 | 0.430 |
| OSATS Time and Motion (R2) | 2.2 ± 0.9 | 2.2 ± 0.9 | 2.2 ± 0.9 | 1.000 |
| OSATS Instrument Handling (R1) | 2.3 ± 1.1 | 2.2 ± 1.2 | 2.4 ± 1.0 | 0.477 |
| OSATS Instrument Handling (R2) | 2.2 ± 0.8 | 2.1 ± 0.7 | 2.3 ± 1.0 | 0.657 |
| OSATS Tissue Exposure (R1) | 2.4 ± 0.9 | 2.4 ± 1.0 | 2.4 ± 0.8 | 0.936 |
| OSATS Tissue Exposure (R2) | 2.7 ± 0.8 | 2.5 ± 0.7 | 2.8 ± 0.8 | 0.362 |
| OSATS Flow of Operation (R1) | 2.5 ± 0.9 | 2.6 ± 1.0 | 2.3 ± 1.0 | 0.499 |
| OSATS Flow of Operation (R2) | 2.8 ± 0.7 | 2.8 ± 0.8 | 2.7 ± 0.7 | 0.805 |
| OSATS Summary (R1) | 2.5 ± 0. | 2.40 ± 1.1 | 2.5 ± 0.9 | 0.538 |
| OSATS Summary (R2) | 2.5 ± 0.8 | 2.5 ± 0.9 | 2.5 ± 0.9 | 1.000 |
| OSATS Cumulative (R1) | 14.3 ± 5.2 | 14.5 ± 5.8 | 14.1 ± 4.8 | 0.970 |
| OSATS Cumulative (R2) | 14.8 ± 4.1 | 14.7 ± 4.2 | 14.8 ± 4.2 | 0.970 |
| **OSATS (R1)** | **2.4 ± 0.9** | **2.4 ± 1.0** | **2.4 ± 0.8** | **0.970** |
| **OSATS (R2)** | **2.5 ± 0.7** | **2.5 ± 0.7** | **2.5 ± 0.7** | **0.970** |
| **Combined OSATS** | **2.4 ± 0.8** | **2.4 ± 0.8** | **2.4 ± 0.7** | **0.791** |

**^Legend supplementary table 12:^** ^Force on tissue in the first and second exam, based on the average of all repetitions per exam (N). Values are median [quartile 1 to quartile 3] or p-value.^

**Supplementary table 13:** Detailed force measurements of the first exam.

| Exam 1 | Total (n = 20) | Intervention group: first robotic simulation, second suture-pads  (n = 10) | Control group: first suture-pads, second robotic simulation  (n = 10) | P-value |
| --- | --- | --- | --- | --- |
| Average force | 0.3 [0.2–0.4] | 0.3 [0.2–0.4] | 0.3 [0.3–0.4] | 0.796 |
| Non-Zero force | 0.4 [0.4–0.6] | 0.4 [0.4–0.6] | 0.5 [0.4–0.6] | 0.853 |
| Maximum force | 5.3 [3.2–7.3] | 5.6 [3.0–7.1] | 4.7 [2.9–8.1] | 0.912 |
| Maximum impulse | 15.4 [7.7–41.7] | 11.2 [7.3–56.2] | 21.0 [10.8–52.0] | 0.353 |

**^Legend supplementary table 13:^** ^Force on tissue in the first exam, based on the average of all repetitions per exam (N). Values are median [quartile 1 to quartile 3] or p-value.^

**Supplementary table 14:** Details on the effect of baseline demographics on surgical performance in the first exam.

| Baseline demographics | Combined OSATS 1 | |
| --- | --- | --- |
|  | Correlation Coefficient | P-value |
| Age | 0.187 | 0.431 |
| Sex | 0.155 | 0.514 |
| Level of residency | 0.326 | 0.160 |
| Open sutures | 0.294 | 0.208 |
| Laparoscopic sutures | 0.345 | 0.137 |
| Laparoscopy training - Simulation | 0.206 | 0.383 |
| Laparoscopy training - Dry-lab | 0.227 | 0.336 |
| Laparoscopy training - Wet lab | 0.022 | 0.927 |
| Laparoscopic procedures - Chief operator | 0.277 | 0.237 |
| Laparoscopic procedures - Assistant | 0.022 | 0.927 |
| Robot training - Simulation | 0.240 | 0.309 |
| Robotic procedures - Assistant | 0.501 | **0.025** |
| Hand dominance | 0.183 | 0.441 |
| Vision correction | 0.192 | 0.416 |
| Learning style | 0.085 | 0.720 |
| Experience with da Vinci | -0.593 | **0.006** |

**^Legend supplementary table 14:^** ^Univariate analysis of the baseline demographics and the combined Objective Structured Assessment of Technical Skills (OSATS) score in the first exam. Values are Correlation Coefficient or p-value.^

# **APPENDIX**

## **Appendix 1.** Survey


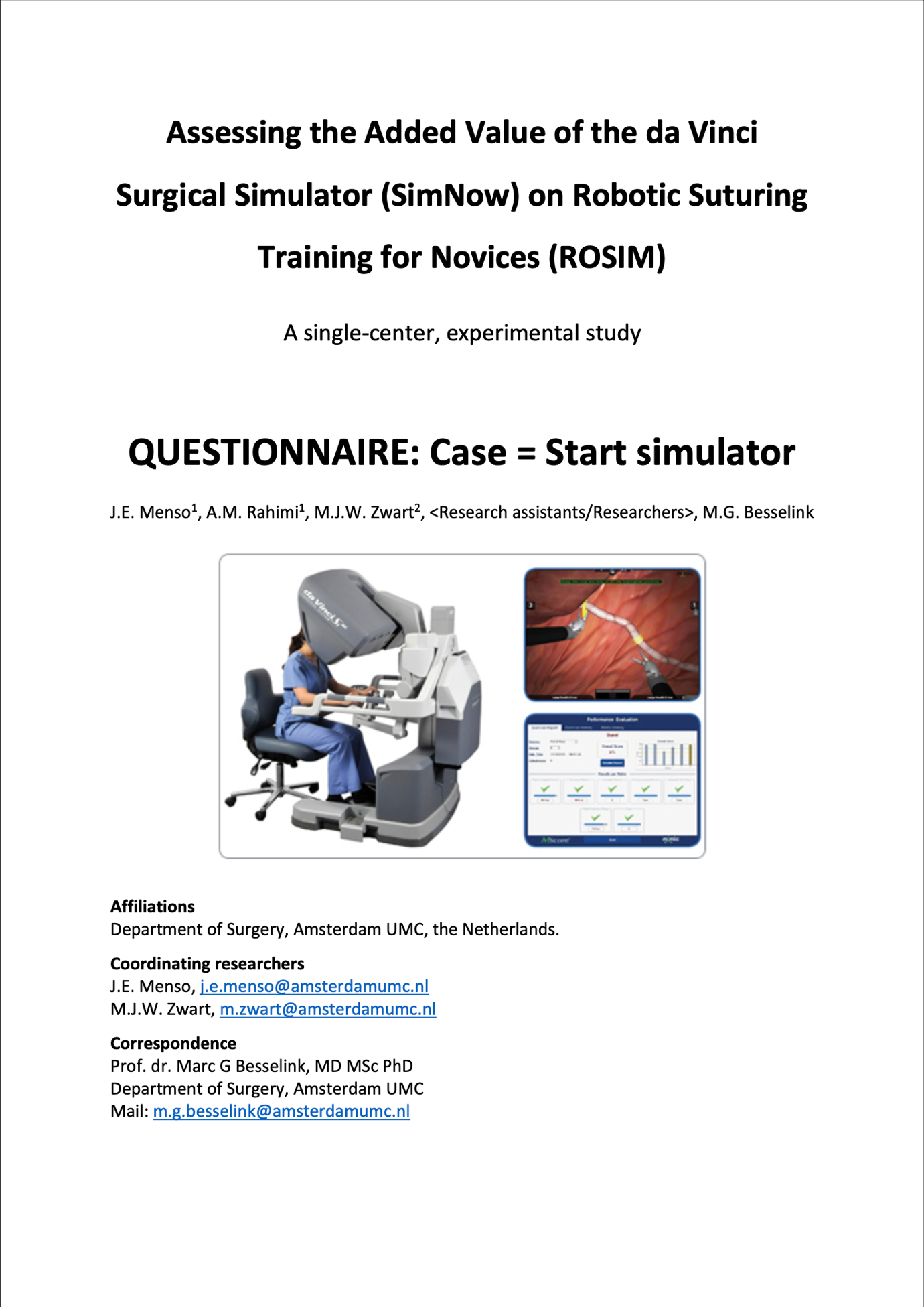


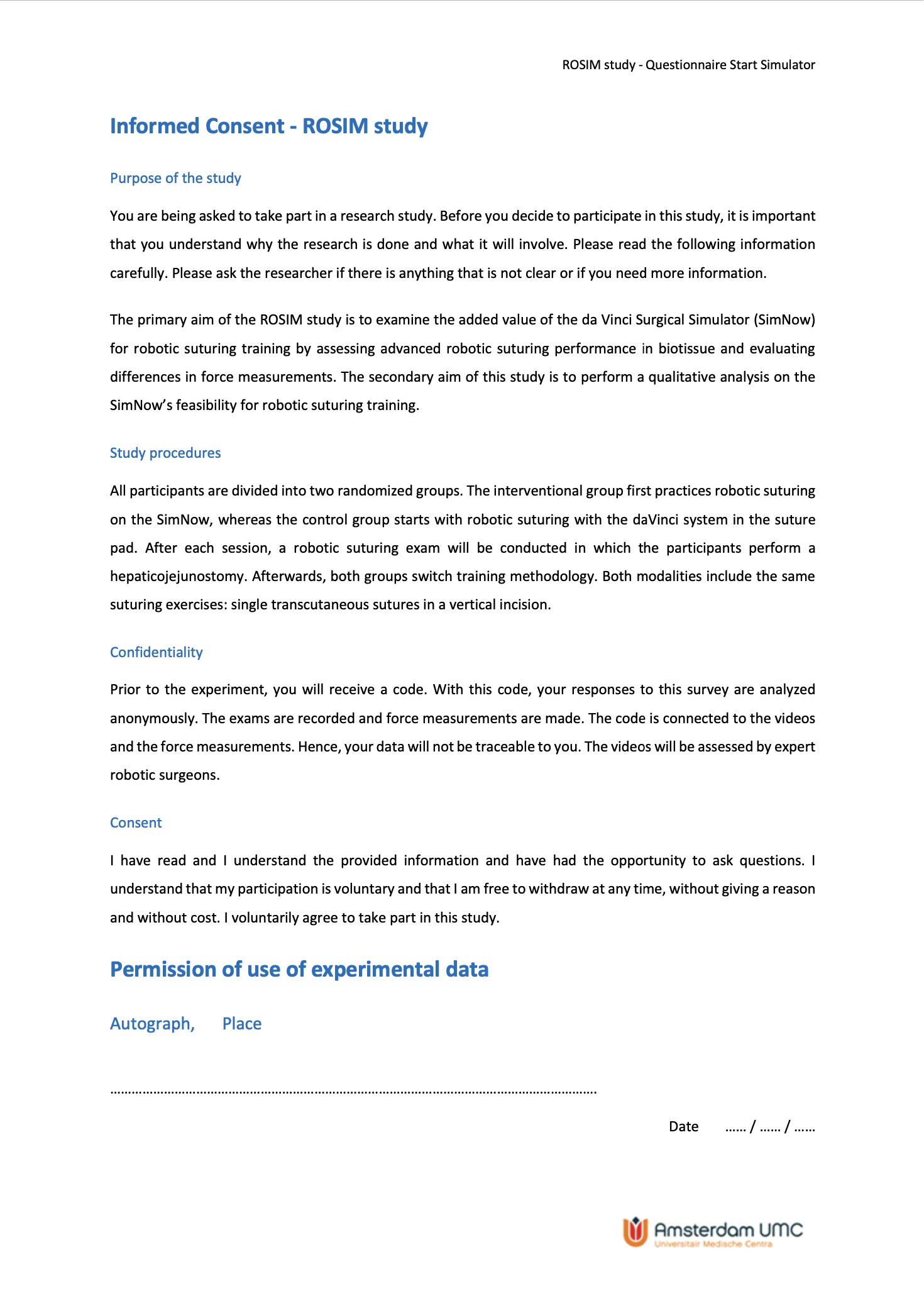


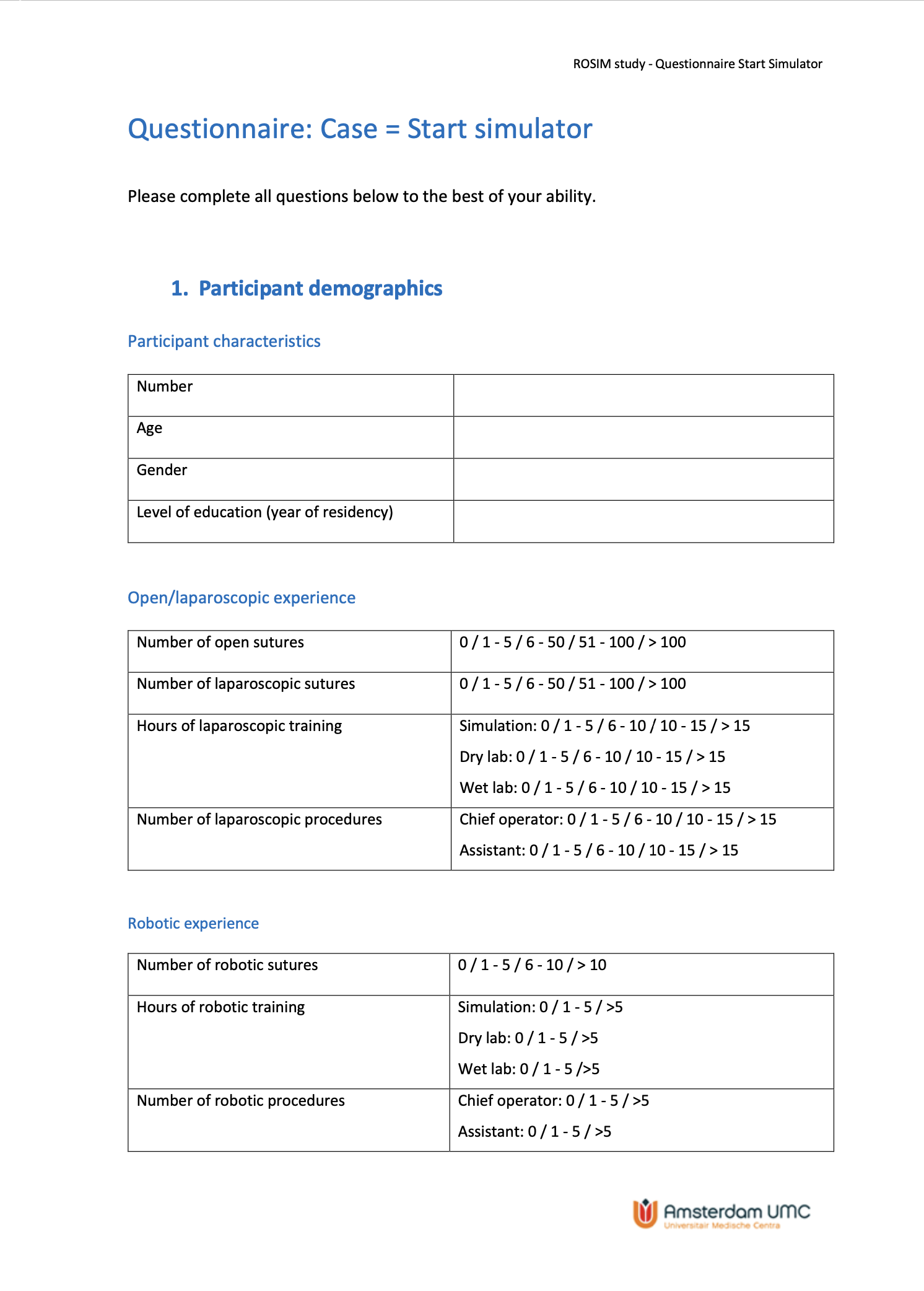


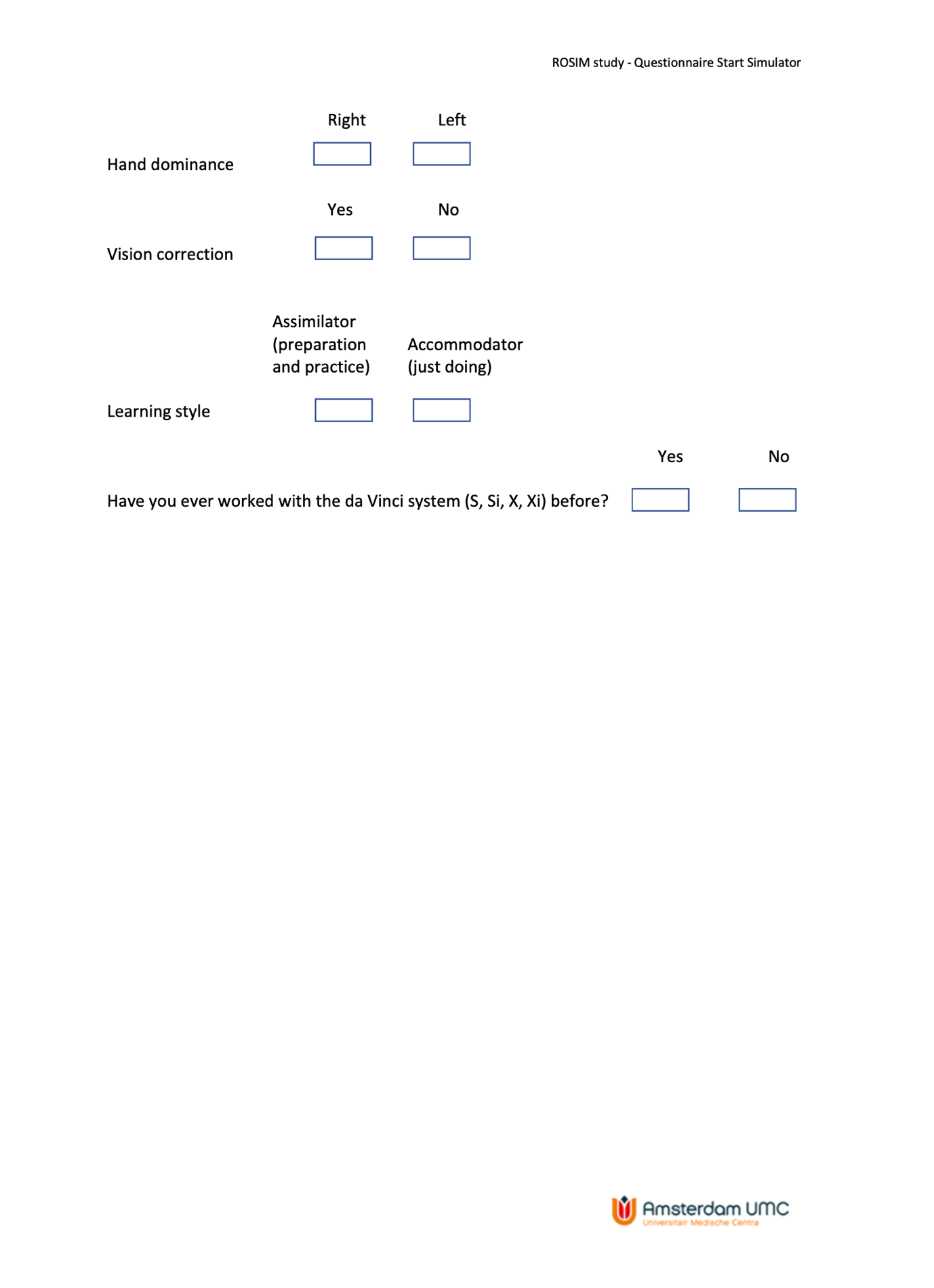


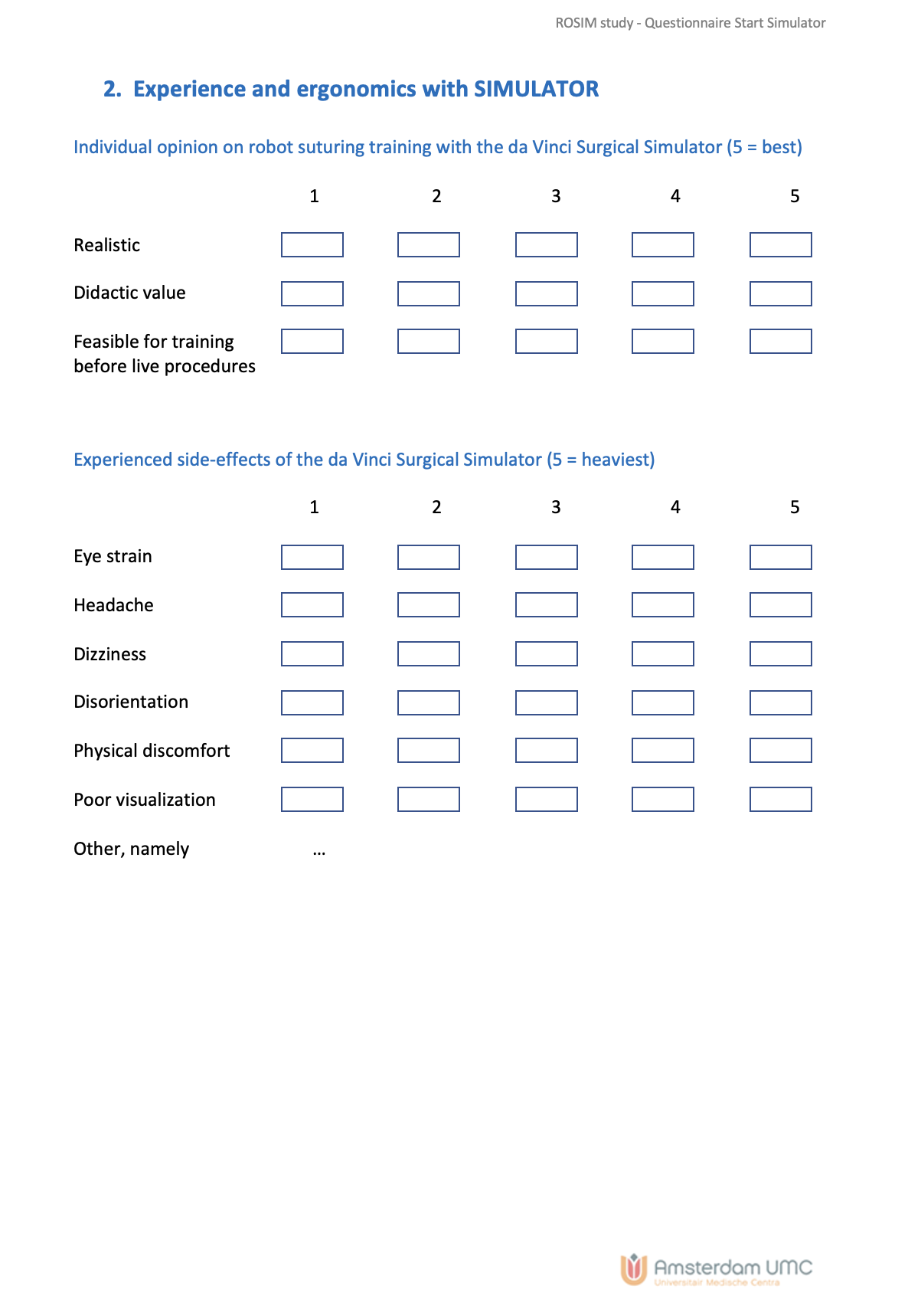


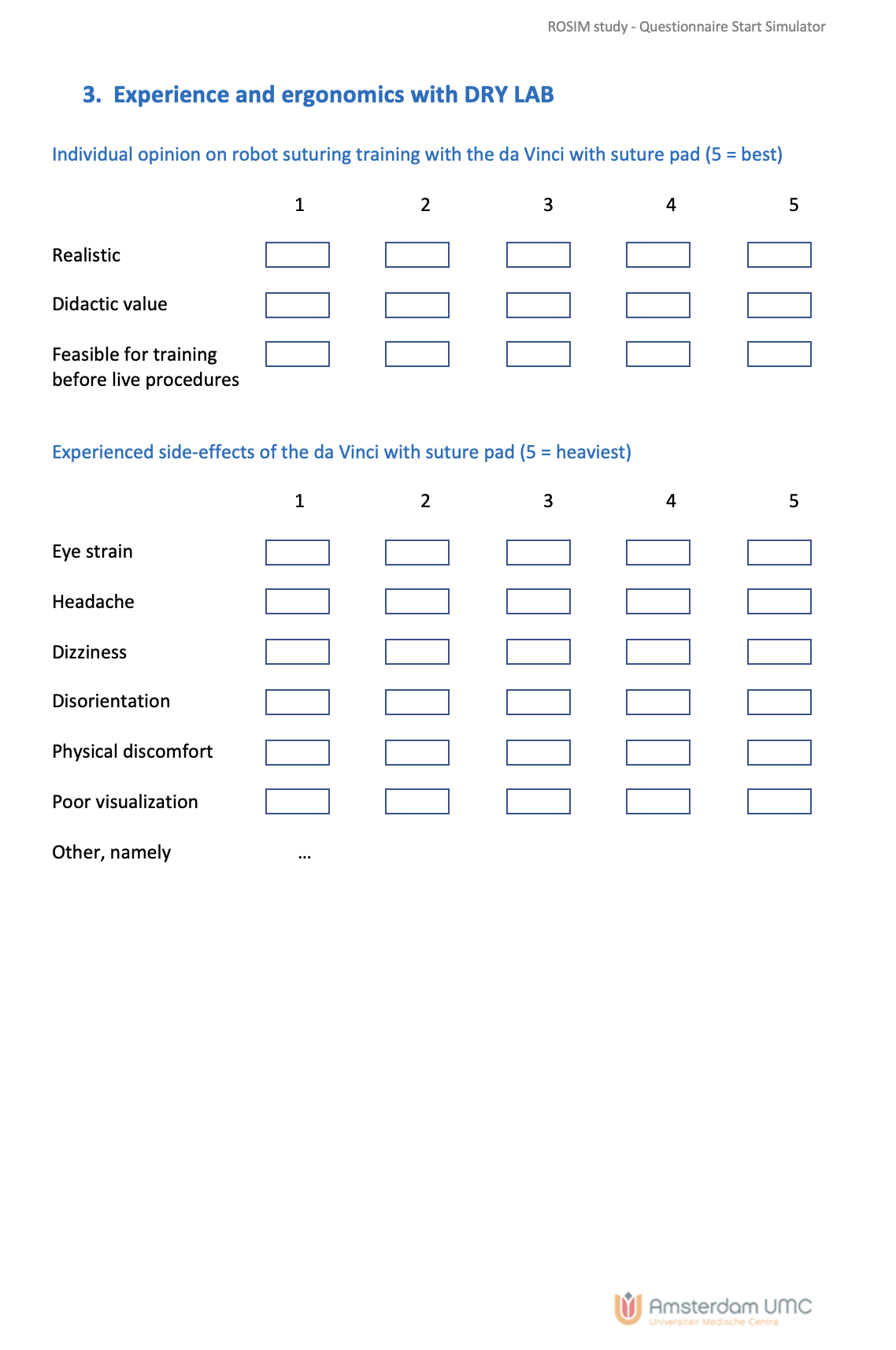


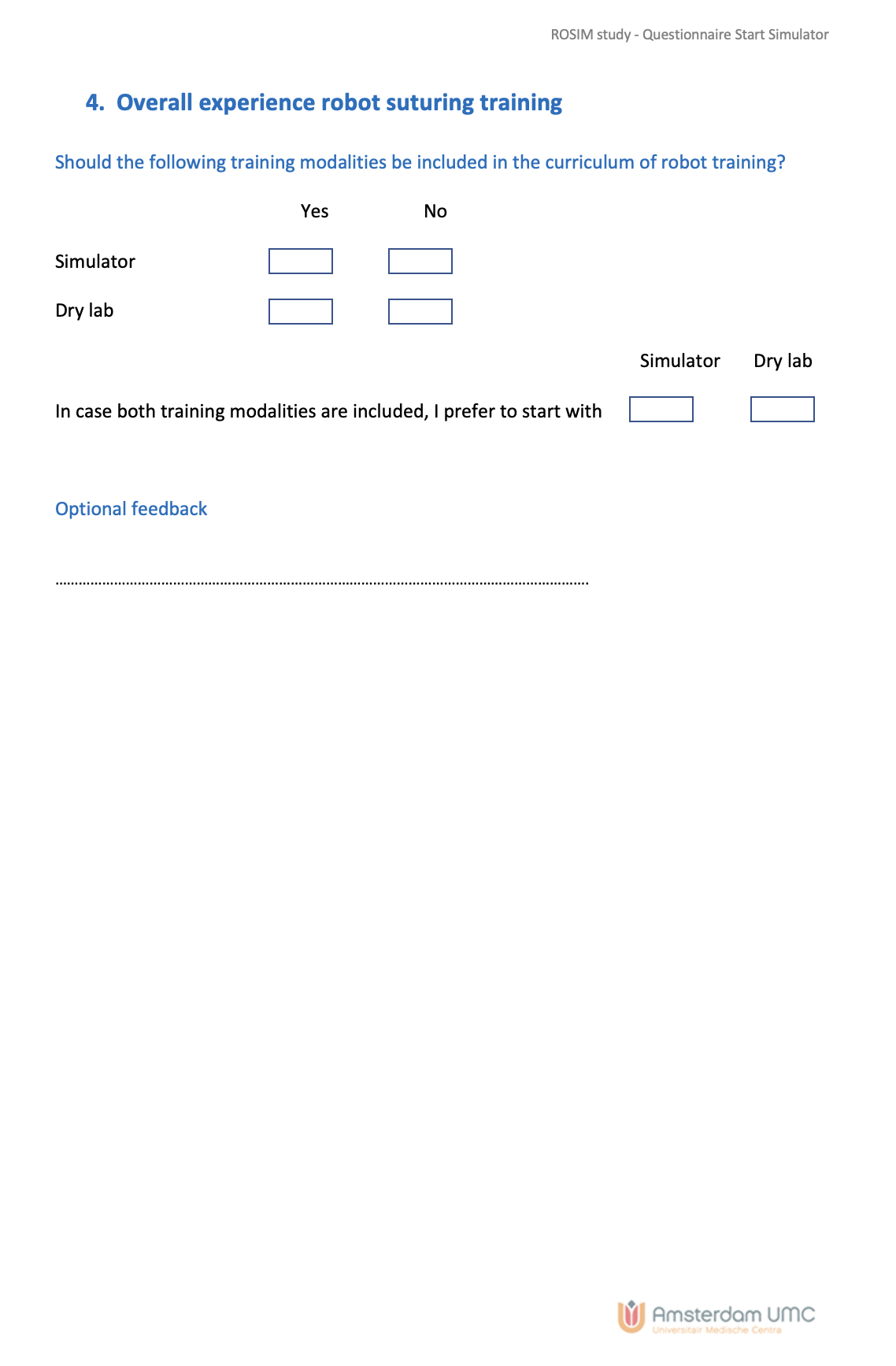

Supplement: Supplementary file 1 — Supplementary file1 (DOCX 3944 KB) [file 464_2024_10914_MOESM1_ESM.docx]
